# Age Dependent Differences in Collagen Alignment of Glutaraldehyde Fixed Bovine Pericardium

**DOI:** 10.1155/2014/189197

**Published:** 2014-09-14

**Authors:** Katie H. Sizeland, Hannah C. Wells, John Higgins, Crystal M. Cunanan, Nigel Kirby, Adrian Hawley, Stephen T. Mudie, Richard G. Haverkamp

**Affiliations:** ^1^School of Engineering and Advanced Technology, Massey University, Palmerston North 4472, New Zealand; ^2^Southern Lights Biomaterials, Palmerston North 4110, New Zealand; ^3^Structural Heart Division, Boston Scientific, Los Gatos, CA 95032, USA; ^4^Australian Synchrotron, Clayton, VIC 3168, Australia

## Abstract

Bovine pericardium is used for heart valve leaflet replacement where the strength and thinness are critical properties. Pericardium from neonatal animals (4–7 days old) is advantageously thinner and is considered as an alternative to that from adult animals. Here, the structures of adult and neonatal bovine pericardium tissues fixed with glutaraldehyde are characterized by synchrotron-based small angle X-ray scattering (SAXS) and compared with the mechanical properties of these materials. Significant differences are observed between adult and neonatal tissue. The glutaraldehyde fixed neonatal tissue has a higher modulus of elasticity (83.7 MPa) than adult pericardium (33.5 MPa) and a higher normalised ultimate tensile strength (32.9 MPa) than adult pericardium (19.1 MPa). Measured edge on to the tissue, the collagen in neonatal pericardium is significantly more aligned (orientation index (OI) 0.78) than that in adult pericardium (OI 0.62). There is no difference in the fibril diameter between neonatal and adult pericardium. It is shown that high alignment in the plane of the tissue provides the mechanism for the increased strength of the neonatal material. The superior strength of neonatal compared with adult tissue supports the use of neonatal bovine pericardium in heterografts.

## 1. Introduction

Heart valve leaflet replacement with bovine pericardium is an established practice [1] using either adult or calf pericardium [2] and may be performed percutaneously [3]. It is essential that the mechanical strength and performance of the material are adequate for a long life in service [4]. Greater understanding of the properties of these materials and the structural basis for these properties is important for improving the serviceability of these replacements.

Pericardium is a fibrous collagen extracellular matrix material with structural similarities to skin and other tissues. The structure of these collagenous tissues can be characterized by small angle X-ray scattering (SAXS) to yield, for example, quantitative measures of fibril orientation and fibril D-spacing [5–7]. While other methods have been used to study collagen fibril orientation including polarized light microscopy [8], reflection anisotropy [9], small angle light scattering [10], confocal laser scattering [11], Raman polarisation [12], and anisotropic Raman scattering [13], synchrotron based SAXS has the advantage of excellent nonsubjective quantification combined with good spatial resolution.

The fact that there is a function-structure relationship between collagen alignment and mechanical strength is well known [14]. The orientation of collagen measured edge on (alignment in plane) has been shown in bovine and ovine skin to be correlated with strength [15, 16]. This correlation extends across a range of mammal species with a strength range of over a factor of five [17]. It is the three-dimensional orientation that is important: simply taking an observation of the fibril orientation normal to the surface of the tissue is not very helpful. Instead, it is necessary to measure the orientation of the fibrils through the thickness of the skin to determine the extent to which they cross between the top and the bottom of the skin layer [17].

The orientation of collagen in pericardium heterograft materials for heart valve leaflets has been shown to affect the stiffness during flexing [18]. In ovine and bovine skin, the orientation of the fibrils in the skin influences the mechanical properties [16, 17].

We have previously found that pericardium from neonatal calves (4–7 days old) has superior properties for potential application for heart valve repair [19]. Although both adult and calf bovine pericardia are used in heart valve repair, neonatal pericardium has not yet been used for heart valve manufacture. The greater tensile modulus of neonatal pericardium compared to that of adult pericardium may enable the thinner neonatal tissue to be used. This would allow a smaller introducer size for percutaneous heart valves. This makes the application of these heart valves possible through diseased femoral arteries which may have reduced diameters [20]. Glutaraldehyde crosslinked pericardium continues to be the material of choice for heart valve manufacturers and developers. There are several devices on the market and more devices currently in clinical trial that use glutaraldehyde treated tissues.

It is known that collagen tissue properties change with age. Differences have been shown in the thermal stability of tendon collagen between steers aged 24–30 months and bulls aged 5 years and this has been attributed to increased level of maturity and thermally stable crosslinks [21]. Glycation of collagen increases with age and can lead to differences in mechanical properties of the collagen. It has been shown to increase stiffness in connective tissues [22] and collagen gels [23] and increase brittleness in bones [24]. Porcine extracellular matrix scaffolds derived from small intestinal submucosa of younger animals and used for* in vivo* remodeling have been studied previously. They were associated with a more constructive, site appropriate, tissue remodeling response than scaffolds derived from older animals [25]. However, specific physical factors causing this difference were not identified.

It has also been found that tissue strength varies with collagen fibril diameter. Larger diameter collagen fibrils are present in stronger tissue. In human aortic valves, the collagen fibril diameter depends on whether the fibrils are from regions of high stress or low stress: larger diameter fibrils (in areas of lower fibril density) result from high stress, suggesting that these larger diameter fibrils provide increased strength [26]. Similarly, for mouse and rat tendon, fibril diameters increase with loading [27, 28]. It is proposed that this is due to the extra mechanical load placed on the tendons on the exercising animals (due to their higher activity levels) stimulating fibril thickening [28]. In bovine leather, fibril diameter is found to be only weakly correlated with strength [29].

The size distribution of the fibril diameter has also been found to change with age. Fetal tissue has been found to have a unimodal distribution with smaller collagen fibril diameters, whereas older tissue has larger fibrils and may have a unimodal or bimodal size distribution depending on the tissue type and animal [30]. In studies of equine digital flexor tendons, fibril diameter decreases with exercise, suggesting weakening of tendon with exercise (i.e., fatter fibril is stronger). Unusually, the fibril diameter in these tendons decreases with age, and this is associated with the decrease in strength [31, 32].

In the percutaneous delivery of heart valves, the size of the device when folded for delivery is important. Devices made from adult bovine pericardium or porcine pericardium typically require a size 18 F to 25 F catheter (7.0–8.4 mm) [33]. This size is in part dictated by the thickness of the pericardium that is used in the valve, with thicker material folding into a larger diameter device for insertion. A study of 79 patients with peripheral arterial disease found that occluded femoral arteries had an average internal diameter of 4.5 ± 1.4 mm with 12 below 3.5 mm (11 F on the French catheter scale) [20]. These occluded arteries are significantly smaller than the folded heart valves resulting in difficulties for percutaneous delivery of existing heart valve technology. This provides a motivation to find thinner but sufficiently strong material as a substitute for the existing bovine or porcine pericardium. Neonatal pericardium is one possible option that is investigated here.

The structural differences between neonatal pericardium and adult tissue that give rise to the desirable differences in their physical properties have not been adequately investigated. This study investigates and compares the collagen fibril structure of neonatal and adult bovine pericardium using SAXS. Specifically, the fibril orientation and the fibril diameter are examined. The use of SAXS at a modern synchrotron facility allows analysis of a small area (250 × 80 *μ*m), enabling quantification of fibril orientation edge on in relatively thin pericardium tissues, a process that is difficult to achieve by other methods.

## 2. Methods

**Figure 1:**
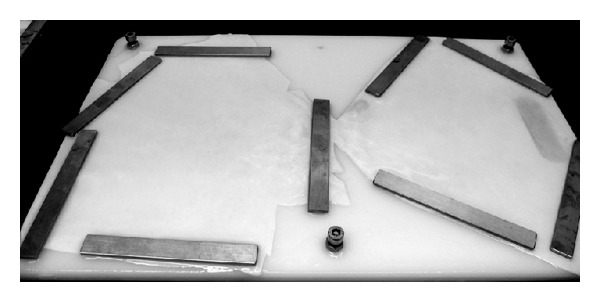
Flattened pericardium, after cutting, held with weights around the edge.

Pericardia were selected from 10 adult (18–24 months old) and 10 neonatal (4–7 days old) cattle. The fresh pericardia (less than 72 hours postmortem and typically 48–72 hours postmortem) were washed several times in PBS buffer (pH 7.4 ± 0.2, 0.01% NaCl). Adult tissue was typically processed closer to 48 hours postmortem while neonatal tissue was processed closer to 72 hours postmortem due to the logistics of obtaining the samples. The tissue was stored at 4–7°C from harvest until the start of washing. Washing in PBS buffer took place at room temperature. The pericardium was then cut and flattened into a “butterfly” shape and held flat with weights around the edge (Figure 1) with care taken to ensure that there were no air bubbles trapped beneath the material.

Treatment with glutaraldehyde was performed in several stages at room temperature. First, the flat, weighted pericardium was immersed in a tray of 0.625% glutaraldehyde (in PBS buffer) for 30 minutes. The second stage was immersion in fresh 0.625% glutaraldehyde (in PBS buffer) for 48 hours with the weights removed. For the third stage the solution was changed for fresh 0.625% glutaraldehyde (in PBS buffer) and maintained for a further 48 hours. After this treatment, coupons of 90 mm × 140 mm were cut from the centre of each side of the butterfly. The pericardium was stored with enough 0.625% glutaraldehyde PBS solution to keep the material moist until being required for other tests. The time between this stage and mechanical measurements varied between 1 and 7 days and for the SAXS measurements between 1 and 3 weeks. For the SAXS analysis, strips were cut in two directions perpendicular to each other from the centre of coupons. Replicates of each sample were prepared.

The thickness was measured with callipers using a light and consistent force.

The elastic modulus and ultimate tensile strength were measured uniaxially using an Instron tensile tester on strips of material.

Histological cross-sections stained with picrosirius red to highlight the collagen were recorded under cross-polarized light on one sample of neonatal pericardium and one of adult pericardium [34].

**Figure 2:**
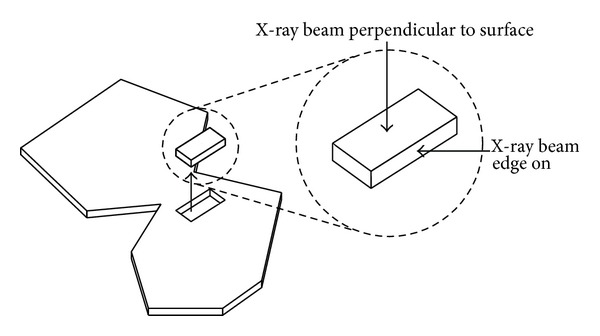
Directions of X-ray analysis of samples.

In preparation for SAXS analysis, the pericardia were removed from the glutaraldehyde solution in which they had been stored. After soaking for at least 1 hour in buffered saline solution, the strips were mounted in 7 *μ*m thick Kapton tape to prevent drying during analysis (i.e., retain them in a wet state). The X-ray beam was directed either through the sample perpendicular to the flat surface or through one of two edge mounted samples. This meant that spectra were recorded in each of the three orthogonal directions through the tissue for each sample (Figure 2). For the edge-analysed samples, it was necessary to brace the tissue against a stiff plastic strip mount to prevent the pericardium from folding or twisting during analysis and to ensure that there was only one layer of the sample in the path of the X-ray beam throughout the travel of the beam. All diffraction patterns were recorded at room temperature.

Diffraction patterns were recorded on the Australian Synchrotron SAXS/WAXS beamline, utilizing a high-intensity undulator source. Energy resolution of 10^−4^ (e.g., 1 × 10^−4^ Å for 1 Å radiation) was obtained from a cryocooled Si(111) double-crystal monochromator and the beam size (FWHM focused at the sample) was 250 × 80 *μ*m, with a total photon flux of about 2 × 10^12^ ph·s^−1^. All diffraction patterns were recorded with an X-ray energy of 12 keV using a Pilatus 1 M detector with an active area of 170 × 170 mm and a sample-to-detector distance of 3371 mm. Exposure time for diffraction patterns was 1 s and data processing was carried out using the SAXS15ID software [35].

The orientation index (OI) is used to give a measure of the spread of microfibril orientation (an OI of 1 indicates the microfibrils are parallel to each other; an OI of 0 indicates the microfibrils are randomly oriented). OI is defined as (90° − OA)/90°, where OA is the minimum azimuthal angle range that contains 50% of the microfibrils. This was based on the method of Sacks for light scattering [10] but converted to an index [15], using the spread in azimuthal angle of, typically, the sixth order peak at approximately 0.055–0.059 Å^−1^. This peak was selected as it is one of the most intense diffraction peaks. The peak area is measured, above a fitted baseline, at each azimuthal angle.

Fibril diameters were calculated from the SAXS data using the Irena software package [36] running within Igor Pro. The data were fitted at the wave vector,* Q*, in the range of 0.01–0.04 Å^−1^ and at an azimuthal angle which was 90° to the long axis of most of the collagen fibrils. This angle was selected by determining the average orientation of the collagen fibrils from the azimuthal angle for the maximum intensity of the D-spacing diffraction peaks. The “cylinderAR” shape model with an arbitrary aspect ratio of 30 was used for all fittings. We did not attempt to individually optimize this aspect ratio and the unbranched length of collagen fibrils may in practice have a length that exceeds an aspect ratio of 30.

## 3. Results

### 3.1. Histology

**Figure 3:**
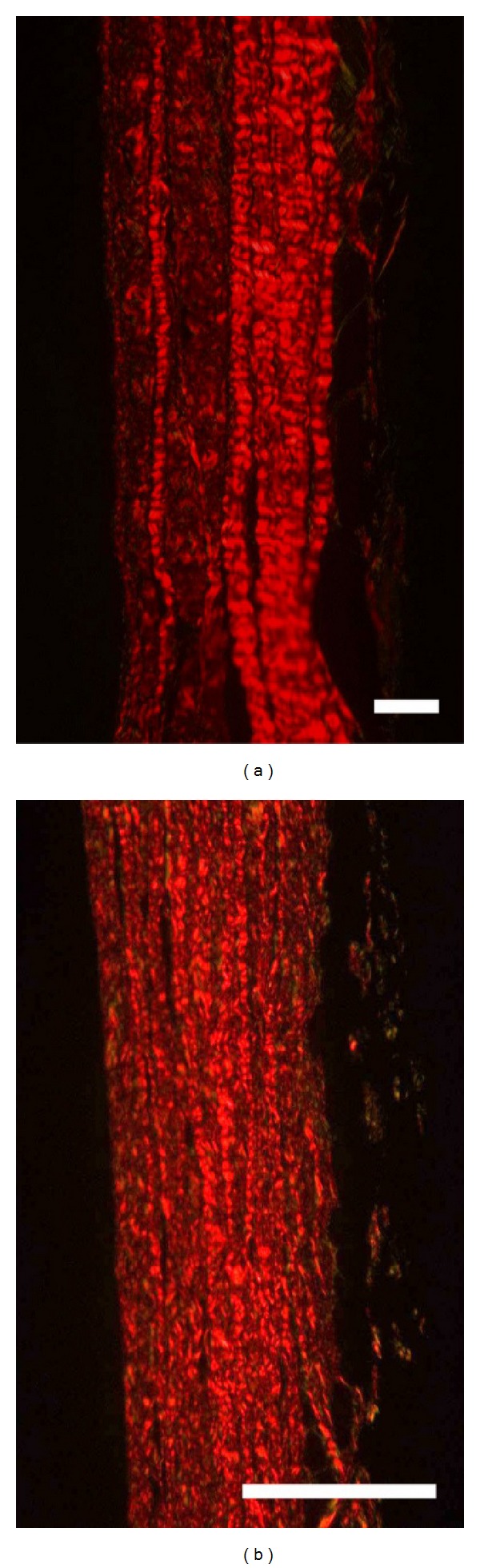
Pericardium stained with picrosirius red and imaged through cross-polarized light to highlight collagen: (a) adult pericardium; (b) neonatal pericardium. The parietal side is toward the left and the fibrous side toward the right of each image. Scale bar is 0.1 mm.

Pericardium stained with picrosirius red highlights differences in the inner and outer layers of the parietal pericardium with more pronounced and larger collagen fibres in the fibrous side of the tissue (Figure 3). Differences are apparent between adult and neonatal pericardium with the adult pericardium more clearly differentiated into two layers. The layer on the fibrous side showed a strongly differentiated collagen fibre structure compared with the parietal side of the adult pericardium. By comparison, the juvenile pericardium has less differentiation through its thickness.

### 3.2. Thickness

The average thickness was 0.36 (*σ* = 0.03) mm for adult pericardium and 0.12 (*σ* = 0.006) mm for neonatal pericardium. The neonatal is therefore one-third of the thickness of the adult.

### 3.3. Mechanical Properties

The elastic modulus of glutaraldehyde fixed neonatal bovine pericardium at both small strain (<20%) and large strain is found to be very much greater than for glutaraldehyde fixed adult pericardium (Table 1).

The ultimate tensile strength is also greater for glutaraldehyde fixed neonatal pericardium than for adult pericardium (Table 1). The strength measured here (standard deviation in parentheses) of 19.1 (*σ* = 2.2) MPa for adult and 32.9 (*σ* = 4.1) MPa for neonatal pericardium is less than that reported previously for unfixed bovine pericardium of 25–29 MPa and of unfixed porcine pericardium of 22-23 MPa [37, 38] but greater than that reported in a different study for calf pericardium at 6–9 months of 11.5 ± 4.6 MPa [39]. We report the ultimate tensile strength as a modulus, that is, tissue property per cross-sectional area. The absolute strength of the glutaraldehyde treated adult pericardium is a little higher than the neonatal. However, it is noted that the neonatal pericardium is one-third of the thickness of adult pericardium. The strain at failure is much less for neonatal pericardium than for adult pericardium. This reflects the higher elastic modulus of the neonatal material compared with adult pericardium and high strength (Table 1).

The main objective of this work is to determine why the neonatal pericardium has a higher ultimate tensile strength and a higher tissue modulus. For this purpose the SAXS measurements were used to investigate the structures of these two materials.

### 3.4. SAXS Measurements

**Figure 4:**
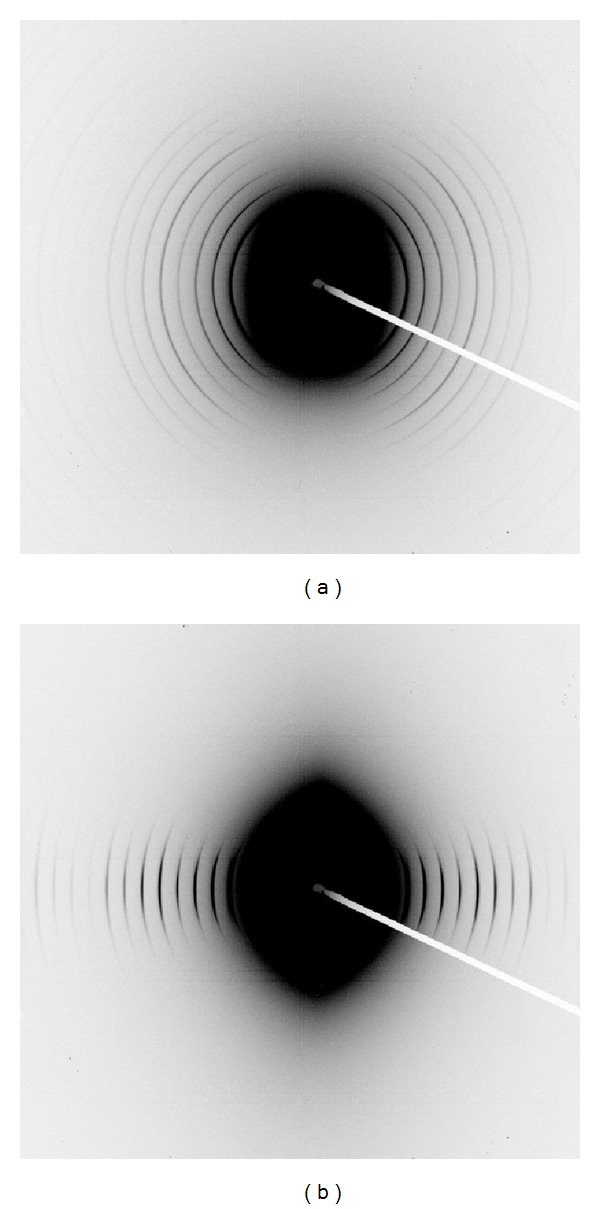
SAXS spectra of pericardium. (a) A poorly oriented tissue; (b) a highly oriented tissue.

**Figure 5:**
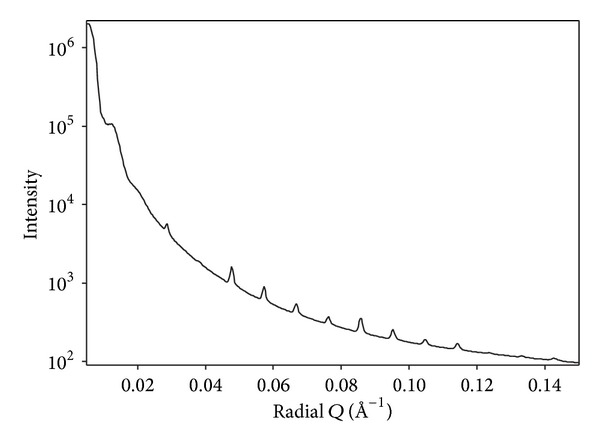
SAXS profile of an example bovine pericardium integrated around all azimuthal angles. The sharp peaks due to collagen D-spacing of various orders are visible (order 5 is just below 0.05 Å^−1^, order 6 at just below 0.06 Å^−1^, etc.).

SAXS patterns of pericardium show clearly the diffraction from collagen fibrils. Two selected SAXS images are shown illustrating a nearly isotropic sample (Figure 4(a)) and a highly oriented sample (Figure 4(b)). The angular position of the bands or rings is due to the D-spacing of the collagen fibrils. The integrated intensity of such a pattern (Figure 5) enables the position of each peak of different order to be accurately measured.

**Figure 6:**
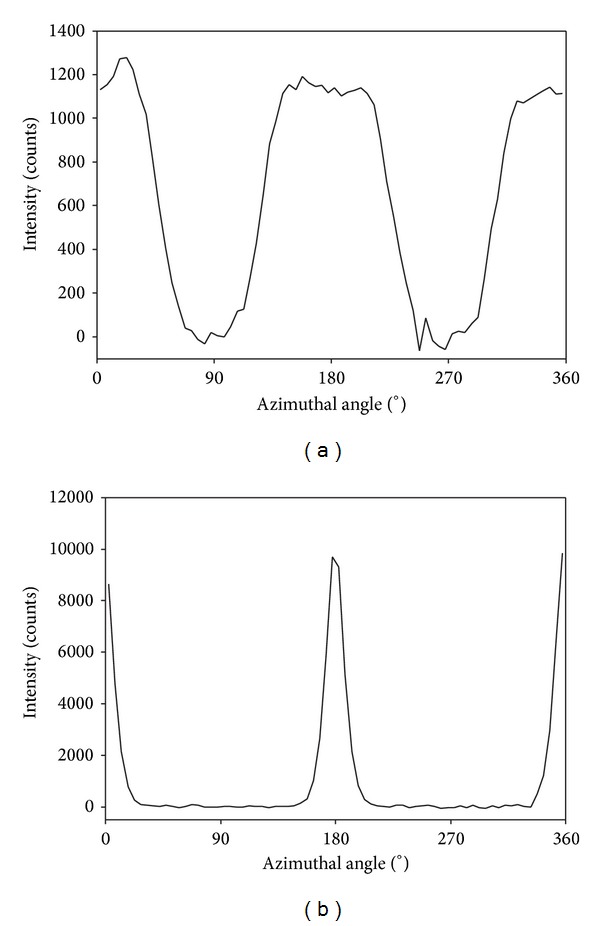
Plots of the intensity of a selected collagen peak at varying azimuthal angles for bovine pericardium samples. (a) A poorly aligned tissue; (b) a highly aligned tissue. The central peak at 180° (and other peaks at 0 and 360°) is the variation in intensity of collagen D-spacing whereas the lower peaks at 90° and 270° are due to the scattering from the thickness of the fibrils and fibril bundles.

To obtain a quantitative measure of the orientation of the fibrils, the variation in intensity of one of these collagen peaks is plotted as a function of azimuthal angle (Figure 6). It is the width of the peak centered at 180° that reflects the orientation index (OI).

### 3.5. Orientation

The collagen fibril OI measured perpendicular to the surface of the pericardium is very small, indicating a highly isotropic arrangement of fibrils in this direction. There is no statistical difference in alignment between adult tissue (OI = 0.020) and neonatal tissue (OI = 0.071) (*t*-statistic = 0.794, *P* = 0.43) (Table 2).

In contrast to the perpendicular measurements, edge on, the fibrils are more oriented and have a higher OI. The fibrils are therefore approximately in isotropic layers stacked one upon the other. However, there are marked differences between the neonatal and the adult pericardium tissues, and these differences are most noticeable in the degree with which these layers intertwine with each other.

Edge on, the adult pericardium tissue has a significantly lower OI than the neonatal tissue measured in both the vertical and the horizontal directions. Measured in the vertical direction, the adult has an OI of 0.581 (*σ* = 0.051) compared with the neonatal OI of 0.800 (*σ* = 0.031). These results are significantly different (*t*-statistic = 21.5, *P* < 0.0001). Measured in the horizontal direction, the OI of adult pericardium is 0.669 (*σ* = 0.032) and the OI of neonatal pericardium is 0.763 (*σ* = 0.106). This shows a significant difference between the two materials (*t*-statistic = 4.4, *P* < 0.0001). In other words, the fibrils in the neonatal tissue are significantly more aligned within the plane of the tissue than those in the adult tissues.

### 3.6. Fibril Diameter

No statistically significant difference was found in the collagen fibril diameter between neonatal and adult bovine pericardium. Fitting a cylinder model to the SAXS data the adult group (*n* = 39) gave a mean (with standard deviation) of 47.7 (*σ* = 3.0) nm while the neonatal group (*n* = 39) gave 48.4 (*σ* = 4.5) nm. Comparing the two sample sets there was no significant difference between the neonatal and the adult pericardium (*t*-statistic = −0.85, *P* = 0.40).

### 3.7. SAXS Cross-Sections

Pericardium tissue is known to vary throughout its thickness and variation in structure is visible in the histological sections (Figure 3). We have measured the variation in OI through cross-sections of glutaraldehyde fixed pericardium for neonatal and adult tissue.

**Figure 7:**
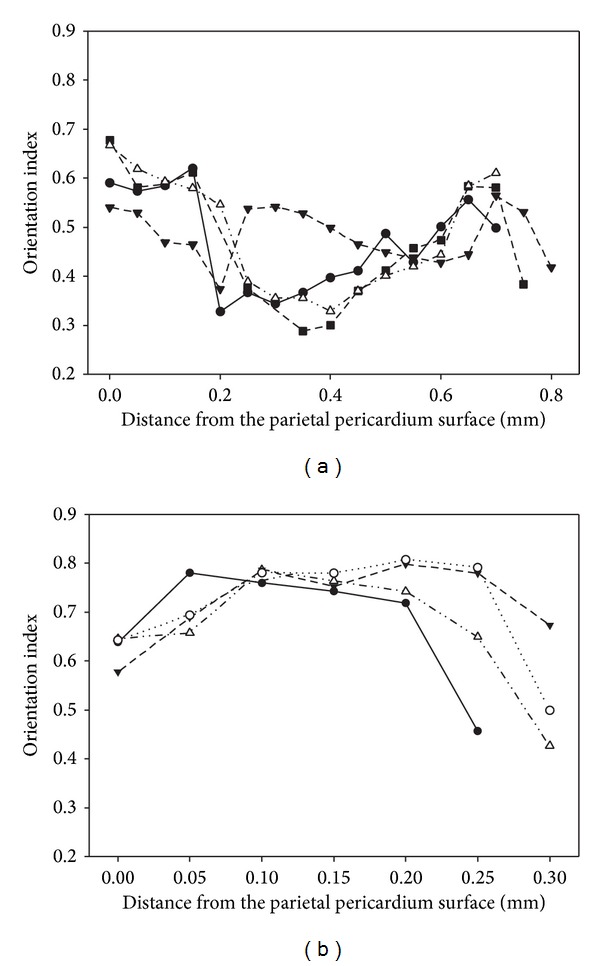
Variation of orientation index through the thickness of glutaraldehyde fixed pericardium: (a) adult; (b) neonatal. Each figure shows two profiles for each of the two samples.

The OI measured through the thickness of glutaraldehyde fixed pericardium does not show a general change from one side to the other (Figure 7). Ovine and bovine leather show a similar flat profile of OI with skin depth [15, 16]. The flat profile between the two halves of the pericardium is somewhat surprising here as the histology shows two distinct layers. The variability in OI across the sample appears to be larger for the adult than for the neonatal tissue. We propose that highly aligned collagen fibrils are responsible for high strength and that varied alignment spreads weakness through the thickness of a material. Variability in alignment in the adult pericardium is thus expected to lower the tensile strength of the adult pericardium.

## 4. Discussion

Adult and neonatal glutaraldehyde fixed pericardium are both useful for constructing bioprosthetic heart valves. However, there are clear differences to be found between the collagen structures in these tissues of different age. These differences are reflected in the orientation of the fibrils.

There was a significant difference observed in the OI between neonatal and adult bovine glutaraldehyde fixed pericardium. For tissue measured edge on, there is a higher OI in the neonatal pericardium than in the adult pericardium. This indicates that collagen fibrils are more aligned in the plane of the tissue in the neonatal pericardium. It is in this direction that the main stresses are applied to pericardium under the elastic deformation during normal heart function. Recent studies of human skin reported a difference in SAXS patterns of young and aged skin (no ages given). The intensity of the collagen diffraction peaks and level of anisotropy were both reported to vary. The aged skin has more intense diffraction peaks and is less anisotropic; that is, it has a lower OI [40], showing a preferential fibril orientation in young individuals that is lost with age. These measurements were taken perpendicularly to the skin. The data presented here for bovine pericardium also shows less anisotropy in the older pericardium. However, this difference is most apparent in the edge-on measurements rather than the perpendicular ones.

It might be expected that the maximum strength of a tissue composed of collagen would be along the direction in which the collagen fibrils are arranged. Thus, when the collagen is more aligned in the direction in which force is applied the tissue will be stronger. A correlation which supports this concept has been observed for ovine and bovine skin of varying strengths and skin from a range of mammals. In those studies, a higher OI (in the plane of the tissue, but not normal to the plane) was correlated with higher tear strength [15, 17]. Therefore the higher OI in edge-on measurements of neonatal pericardium should indicate improved tear strength of this tissue in comparison with adult pericardium. This is indeed observed in tensile testing of neonatal and adult bovine pericardium. Neonatal pericardium demonstrated a markedly higher modulus of elasticity and a higher tensile strength than adult pericardium [19].

Extending and supporting this concept further, myxomatous and healthy mitral valve leaflets from dogs have been reported to have different degrees of fibril orientation. Myxomatous tissue was found to become less aligned [41] which is a possible contributor to the diminished mechanical performance of this tissue although other changes are present too.

Measured perpendicularly, the OI for both adult and neonatal tissue is very low. The tissues are therefore rather isotropic measured from this direction. This is a very useful property as it may enable material to be cut in any direction from the pericardium for use in heart valve leaflets with minimal differences in mechanical performance.

We believe that the greater alignment in the plane of the tissue is the structural basis for the superior mechanical performance of glutaraldehyde fixed neonatal bovine pericardium. A relationship between fibre alignment and tensile strength has been modelled previously. Strength was found to be due to the sum of the components of the fibrils that lie in the direction of force in addition to a component due to the other matrix materials [42]. This model has been applied to just the measured fibrous collagen, neglecting the contribution from other matrix components. A model orientation index is derived which we will call OI′ to distinguish it from the experimentally measured OI [43, 44]:

(1) $$\begin{array}{c} {\text{OI}}^{\prime }=\frac{\int_{0}^{2\pi }\int_{0}^{\pi /2}\cos^{4}\theta F\left(\theta ,\phi \right)d\theta \;d\phi }{\int_{0}^{2\pi }\int_{0}^{\pi /2}F\left(\theta ,\phi \right)d\theta \;d\phi }, \end{array}$$

where *F*(*θ*, *ϕ*) is the angular distribution function where *θ* and *ϕ* are orthogonal. We have previously applied this model to collagen orientation in leather produced from the skins of a selection of mammals. It was found to be valid across a wide range of strengths, that is, greater than a factor of 5 [17].

Some of the apparent nonalignment of fibrils (resulting in a lower OI) could be due to crimp of the collagen fibrils and not only to the variation in whole fibril alignment. However we do not believe this is an important consideration in the materials studied. Crimp is observed in the light microscopy sections (Figure 3) and would contribute to a decrease in the OI in the direction of SAXS measurements taken edge on. In horse tendon crimp has been found to decrease with age [45], as does the tendon strength, and if there were no other changes to the alignment of collagen in the tendons, this would result in an increase in OI with age. This is the opposite of what is observed for the OI change between the neonatal and the adult pericardium. Another consideration which leads us to believe crimp is not an important factor in the measurements reported here is that crimp is generally believed to be associated with strength [46]. Therefore if the OI measured edge on was only due to crimp, this would suggest that the neonatal pericardium, which is stronger, has less crimp and this would contradict the accepted knowledge of the influence of crimp. However, if the differences in OI measured edge on between neonatal and adult pericardium are due to differences in alignment of the collagen fibrils, then this relationship would be in agreement with previous studies on skin (treated to produce leather) where a high degree of alignment is correlated with high strength (and crimp is not present) [15, 17].

We also do not know if these tissues of different age are affected differently by glutaraldehyde treatment. It has been observed that glutaraldehyde treatment lowers the OI of pericardium [47] and it is possible that the neonatal and adult pericardium are affected to a different extent. We are currently investigating this possibility.

We did not find that changes in fibril diameter are responsible for differences in strength between neonatal and adult pericardium. This is a little surprising, as fibril diameter is generally believed to affect strength in tendons and age can result in different fibril diameter with typically older tissue having thicker fibrils. However, in tendons the collagen fibrils are more highly aligned than pericardium so perhaps there is less variation in alignment in tendons and therefore factors other than alignment dominate strength differences. On the other hand, in pericardium (and leather) there is the possibility of significant variation in alignment and therefore alignment may be able to dominate the strength-structure relationship with fibril diameter being a less important factor [29].

The glutaraldehyde treated adult material had a greater modulus of elasticity and greater ultimate tensile strength than the glutaraldehyde treated neonatal material, while the neonatal material was significantly thinner. This suggests that there may be an advantage in the use of this material in applications such as heart valve leaflets for percutaneous delivery. A thinner tissue is able to be folded to be inserted in a much smaller sized catheter for aortic valve replacement. By using the thinner, but sufficiently strong, neonatal bovine pericardium, it could be possible to reduce the catheter diameter required for the insertion of the folded valve. This is an important consideration for many patients needing such intervention [20, 33].

What we have not determined is the relationship between the collagen fibril alignments in the glutaraldehyde fixed material and in the native material. Treatment of native tissue results in differences in strength, as shown here and elsewhere [48]. Cross-linking of the collagen fibrils might be expected to have an effect on fibril alignment because the cross-linking would place physical constraints on the fibrils. Therefore variation in the degree to which cross-linking takes place in different tissues might result in a variable change in alignment. This is the subject of a study we are currently undertaking.

## 5. Conclusions

We have shown that glutaraldehyde fixed neonatal pericardium has higher elastic modulus and ultimate tensile strength than adult pericardium. We found, using SAXS, that there are clear differences in the structure of collagen between neonatal and adult bovine pericardium. The alignment of the collagen in the plane of the tissue is greater in the neonatal pericardium. We have described how this gives a structural understanding of the superior mechanical properties of that material. These findings provide a basis for the potential advantages to using neonatal rather than adult bovine pericardium in heterografts.

## Figures and Tables

**Table 1:** Mechanical properties of adult and neonatal glutaraldehyde fixed bovine pericardium.

Test	Adult (*n* = 13)^†^	Neonatal (*n* = 11)^†^	*P* ^†^
Small strain (<0.2) elastic modulus (MPa)	4.8 (2.0)	71.9 (11.6)	<0.0001
Large strain (>0.2) elastic modulus (MPa)	33.5 (3.2)	83.7 (10.6)	<0.0001
Ultimate tensile strength (MPa)	19.1 (2.2)	32.9 (4.1)	0.0050
Strain at failure	0.80 (0.06)	0.48 (0.03)	0.0002

^†^Standard error () and *P* from two-tailed *t*-test comparison of adult and neonatal pericardium.

**Table 2:** Orientation index (OI) for pericardium samples measured perpendicular and edge-on to the surface for samples cut vertically or horizontally from the pericardium.

Direction measured	Animal age	OI	Std deviation	No. of pericardia	No. of measurements
Perpendicular	Adult	0.020	0.096	10	42
Perpendicular	Neonatal	0.071	0.152	10	42
Edge-on vertical	Adult	0.581	0.051	2	52
Edge-on horizontal	Adult	0.669	0.032	2	27
Edge-on vertical	Neonatal	0.800	0.031	2	30
Edge-on horizontal	Neonatal	0.763	0.106	2	27

## References

[1] Nwaejike N, Ascione R (2011). Mitral valve repair for disruptive acute endocarditis: extensive replacement of posterior leaflet with bovine pericardium. *Journal of Cardiac Surgery*.

[2] García Páez JM, Carrera Sanmartín A, Jorge Herrero E (2006). Durability of a cardiac valve leaflet made of calf pericardium: fatigue and energy consumption. *Journal of Biomedical Materials Research A*.

[3] Cribier A, Eltchaninoff H, Tron C (2003). Percutaneous artificial cardiac valves: From animal experimentation to the first human implantation in a case of calcified aortic stenosis. *Archives des Maladies du Coeur et des Vaisseaux*.

[4] Mirnajafi A, Zubiate B, Sacks MS (2010). Effects of cyclic flexural fatigue on porcine bioprosthetic heart valve heterograft biomaterials. *Journal of Biomedical Materials Research A*.

[5] Liao J, Yang L, Grashow J, Sacks MS (2005). Molecular orientation of collagen in intact planar connective tissues under biaxial stretch. *Acta Biomaterialia*.

[6] Purslow PP, Wess TJ, Hukins DWL (1998). Collagen orientation and molecular spacing during creep and stress-relaxation in soft connective tissues. *Journal of Experimental Biology*.

[7] Basil-Jones MM, Edmonds RL, Allsop TF (2010). Leather structure determination by small-angle X-ray scattering (SAXS): cross sections of ovine and bovine leather. *Journal of Agricultural and Food Chemistry*.

[8] Julkunen P, Iivarinen J, Brama PA, Arokoski J, Jurvelin JS, Helminen HJ (2010). Maturation of collagen fibril network structure in tibial and femoral cartilage of rabbits. *Osteoarthritis and Cartilage*.

[9] Schofield AL, Smith CI, Kearns VR (2011). The use of reflection anisotropy spectroscopy to assess the alignment of collagen. *Journal of Physics D: Applied Physics*.

[10] Sacks MS, Smith DB, Hiester ED (1997). A small angle light scattering device for planar connective tissue microstructural analysis. *Annals of Biomedical Engineering*.

[11] Jor JWY, Nielsen PMF, Nash MP, Hunter PJ (2011). Modelling collagen fibre orientation in porcine skin based upon confocal laser scanning microscopy. *Skin Research and Technology*.

[12] Falgayrac G, Facq S, Leroy G, Cortet B, Penel G (2010). New method for raman investigation of the orientation of collagen fibrils and crystallites in the haversian system of bone. *Applied Spectroscopy*.

[13] Janko M, Davydovskaya P, Bauer M, Zink A, Stark RW (2010). Anisotropic Raman scattering in collagen bundles. *Optics Letters*.

[14] Fratzl P, Weinkamer R (2007). Nature's hierarchical materials. *Progress in Materials Science*.

[15] Basil-Jones MM, Edmonds RL, Cooper SM, Haverkamp RG (2011). Collagen fibril orientation in ovine and bovine leather affects strength: a small angle X-ray scattering (SAXS) study. *Journal of Agricultural and Food Chemistry*.

[16] Basil-Jones MM, Edmonds RL, Norris GE, Haverkamp RG (2012). Collagen fibril alignment and deformation during tensile strain of leather: a small-angle X-ray scattering study. *Journal of Agricultural and Food Chemistry*.

[17] Sizeland KH, Basil-Jones MM, Edmonds RL (2013). Collagen orientation and leather strength for selected mammals. *Journal of Agricultural and Food Chemistry*.

[18] Mirnajafi A, Raymer J, Scott MJ, Sacks MS (2005). The effects of collagen fiber orientation on the flexural properties of pericardial heterograft biomaterials. *Biomaterials*.

[19] Cunanan CM, Higgins JJ, Gurazada SN Biomaterials with enhanced properties and devices made therefrom.

[20] Kroger K, Buss C, Goyen M, Santosa F, Rudofsky G (2002). Diameter of occluded superficial femoral arteries limits percutaneous recanalization: preliminary results. *Journal of Endovascular Therapy*.

[21] Willett TL, Labow RS, Aldous IG, Avery NC, Lee JM (2010). Changes in collagen with aging maintain molecular stability after overload: evidence from an in vitro tendon model. *Journal of Biomechanical Engineering*.

[22] Bailey AJ (2001). Molecular mechanisms of ageing in connective tissues. *Mechanisms of Ageing and Development*.

[23] Francis-Sedlak ME, Uriel S, Larson JC, Greisler HP, Venerus DC, Brey EM (2009). Characterization of type I collagen gels modified by glycation. *Biomaterials*.

[24] Leeming DJ, Henriksen K, Byrjalsen I (2009). Is bone quality associated with collagen age?. *Osteoporosis International*.

[25] Sicari BM, Johnson SA, Siu BF (2012). The effect of source animal age upon the in vivo remodeling characteristics of an extracellular matrix scaffold. *Biomaterials*.

[26] Balguid A, Driessen NJB, Mol A (2008). Stress related collagen ultrastructure in human aortic valves-implications for tissue engineering. *Journal of Biomechanics*.

[27] Michna H (1984). Morphometric analysis of loading-induced changes in collagen- fibril populations in young tendons. *Cell and Tissue Research*.

[28] Biancalana A, Veloso L, Gomes L (2010). Obesity affects collagen fibril diameter and mechanical properties of tendons in Zucker rats. *Connective Tissue Research*.

[29] Wells HC, Edmonds RL, Kirby N, Hawley A, Mudie ST, Haverkamp RG (2013). Collagen fibril diameter and leather strength. *Journal of Agricultural and Food Chemistry*.

[30] Parry DAD, Barnes GRG, Craig AS (1978). A comparison of the size distribution of collagen fibrils in connective tissues as a function of age and a possible relation between fibril size distribution and mechanical properties. *Proceedings of the Royal Society of London—Biological Sciences*.

[31] Patterson-Kane JC, Wilson AM, Firth EC, Parry DAD, Goodship AE (1997). Comparison of collagen fibril populations in the superficial digital flexor tendons of exercised and nonexercised Thoroughbreds. *Equine Veterinary Journal*.

[32] Cherdchutham W, Becker CK, Spek ER, Voorhout WE, Van Weeren PR (2001). Effects of exercise on the diameter of collagen fibrils in the central core and periphery of the superficial digital flexor tendon in foals. *American Journal of Veterinary Research*.

[33] Chiam PTL, Ruiz CE (2008). Percutaneous transcatheter aortic valve implantation: assessing results, judging outcomes, and planning trials. the interventionalist perspective. *JACC Cardiovascular Interventions*.

[34] Constantine VS, Mowry RW (1968). Selective staining of human dermal collagen, II: the use of picrosirius red F3BA with polarization microscopy. *Journal of Investigative Dermatology*.

[35] Cookson D, Kirby N, Knott R, Lee M, Schultz D (2006). Strategies for data collection and calibration with a pinhole-geometry SAXS instrument on a synchrotron beamline. *Journal of Synchrotron Radiation*.

[36] Ilavsky J, Jemian PR (2009). Irena: tool suite for modeling and analysis of small-angle scattering. *Journal of Applied Crystallography*.

[37] Páez JMG, Jorge E, Rocha A (2002). Mechanical effects of increases in the load applied in uniaxial and biaxial tensile testing. Part II. Porcine pericardium. *Journal of Materials Science: Materials in Medicine*.

[38] García Páez JM, Jorge E, Rocha A (2002). Mechanical effects of increases in the load applied in uniaxial and biaxial tensile testing: part I. Calf pericardium. *Journal of Materials Science: Materials in Medicine*.

[39] Maestro MM, Turnay J, Olmo N (2006). Biochemical and mechanical behavior of ostrich pericardium as a new biomaterial. *Acta Biomaterialia*.

[40] Cócera M, Rodríguez G, Rubio L (2011). Characterisation of skin states by non-crystalline diffraction. *Soft Matter*.

[41] Hadian M, Corcoran BM, Han RI, Grossmann JG, Bradshaw JP (2007). Collagen organization in canine myxomatous mitral valve disease: an X-ray diffraction study. *Biophysical Journal*.

[42] Bigi A, Ripamonti A, Roveri N, Jeronimidis G, Purslow PP (1981). Collagen orientation by X-ray pole figures and mechanical properties of media carotid wall. *Journal of Materials Science*.

[43] Kronick PL, Sacks MS (1991). Quantification of vertical-fiber defect in cattle hide by small-angle light scattering.. *Connective Tissue Research*.

[44] Kronick PL, Buechler PR (1986). Fiber orientation in calfskin by laser-light scattering or X-ray-diffraction and quantitative relation to mechanical-properties. *Journal of the American Leather Chemists Association*.

[45] Patterson-Kane JC, Firth EC, Goodship AE, Parry DAD (1997). Age-related differences in collagen crimp patterns in the superficial digital flexor tendon core region of untrained horses. *Australian Veterinary Journal*.

[46] Caves JM, Kumar VA, Xu W, Naik N, Allen MG, Chaikof EL (2010). Microcrimped collagen fiber-elastin composites. *Advanced Materials*.

[47] Lee JM, Haberer SA, Boughner DR (1989). The bovine pericardial xenograft: I. Effect of fixation in aldehydes without constraint on the tensile viscoelastic properties of bovine pericardium. *Journal of Biomedical Materials Research*.

[48] Freytes DO, Badylak SF, Webster TJ, Geddes LA, Rundell AE (2004). Biaxial strength of multilaminated extracellular matrix scaffolds. *Biomaterials*.

